# Adaptation or Malignant Transformation: The Two Faces of Epigenetically Mediated Response to Stress

**DOI:** 10.1155/2013/954060

**Published:** 2013-09-26

**Authors:** Aleksandar Vojta, Vlatka Zoldoš

**Affiliations:** Department of Molecular Biology, Faculty of Science, University of Zagreb, Horvatovac 102a, HR-10000 Zagreb, Croatia

## Abstract

Adaptive response to stress is a fundamental property of living systems. At the cellular level, many different types of stress elicit an essentially limited repertoire of adaptive responses. Epigenetic changes are the main mechanism for medium- to long-term adaptation to accumulated (intense, long-term, or repeated) stress. We propose the adaptive deregulation of the epigenome in response to stress (ADERS) hypothesis which assumes that the unspecific adaptive stress response grows stronger with the increasing stress level, epigenetically activating response gene clusters while progressively deregulating other cellular processes. The balance between the unspecific adaptive response and the general epigenetic deregulation is critical because a strong response can lead to pathology, particularly to malignant transformation. The main idea of our hypothesis is the continuum traversed by a cell subjected to accumulated stress, which lies between an unspecific adaptive response and pathological deregulation—the two extremes sharing the same underlying cause, which is a manifestation of a unified epigenetically mediated adaptive response to stress. The evolutionary potential of epigenetic regulation in multigenerational adaptation is speculatively discussed in the light of neo-Lamarckism. Finally, an approach to testing the proposed hypothesis is presented, relying on either the publicly available datasets or on conducting new experiments.

## 1. Common Cellular Response to Stress

Any departure from a narrow window of optimal physical, chemical, or biological parameters represents stress, to which all living systems react in an adaptive manner aiming to restore homeostasis, either by influencing their environment or undergoing internal adaptation which enables the new situation to be tolerated. The repertoire of stress responses, especially at the cellular level, is relatively limited when compared with the number of apparently different sources of environmental stress. The idea of unified pathways responding to multiple related or even seemingly unrelated stressors has already been formulated in the context of the gatekeeper hypothesis [1]. The cellular pathways activated in response to various types of stress are interconnected and share some features found in almost every stress response type: growth arrest, changes in expression patterns, and programmed cell death in case the damage is too extensive to be dealt with effectively.

Damage to the genome, usually in the form of depurination or formation of pyrimidine dimers, elicits a specific repair response mediated by the p53 tumor suppressor and closely connected to other stress-response pathways [2]. This type of response is indispensable in maintenance of genome integrity and thus ubiquitous across different cell types as well as different taxa.

An evolutionary conserved and ancient adaptation pathway is the heat-shock response which, interestingly, activates molecular chaperones not only in response to heat-induced protein unfolding but also to unfolding caused by heavy metal ions, ethanol, or other toxins [3]. The prominent role of this pathway as a general stress response is remarkably well illustrated by the phenomenon of cross-tolerance, where, for example, heat shock induces a response that also protects from oxidative stress [4]. Unsurprisingly, this pathway has a significant role in certain types of cancer [5].

The autophagy response, where proteins and defective organelles are degraded by lysosomes, is stimulated by multiple forms of stress: starvation, hypoxia, reactive oxygen species, DNA damage, protein aggregates, and pathogens [6]. This important component of the integrated stress response has a prominent role in adaptation and survival of tumor cells [7].

Another type of adaptation pathway with a role in cancer is the hypoxia-inducible factor (HIF) response to hypoxia [8], which promotes a stem-cell-like state in some tissues, as well as in malignantly transformed cells. On a physiological level, the hypoxia response is also involved in ischemia and inflammation.

A hub connecting many stress response pathways is the mTOR kinase, itself primarily responsible for nutrient sensing and adaptation to nutrient stress [9]. Its central position links mTOR with balancing of energy levels, amino acids, glucose, oxygen, and growth factors. The importance of stress response integration by mTOR is evidenced by its role in aging and tumor cell resistance to therapy [10].

Perhaps the best illustration for selective regulation of targeted groups of genes in response to stress is the micro RNA (miRNA) mechanism [11], based on the ~22-nucleotide-long noncoding RNA molecules which profoundly influence expression levels by selectively degrading targeted classes of mRNA, where miRNA participates in a complex assembled around the Argonaute protein, that is, the RNA-induced silencing complex (RISC). In response to oxidative stress, nutrient deprivation, DNA damage, or oncogenic stress [12], ~350 types of miRNAs regulate more than 25% of protein-coding genes in a typical mammalian cell, clearly showing that there are multiple targets—whole classes of genes—regulated by a single miRNA molecule.

Translational reprogramming [13] is another universal mechanism which acts at the level of selective recruitment of ribosomes to specific mRNA molecules involved in response to many of the various types of stress: nutrient deprivation or excess, temperature, DNA lesions, and hypoxia.

Multiple related (or even apparently unrelated) sources of stress elicit unified responses at the cellular level—activation of appropriate “effectors”, which are sets of genes encoding components of the appropriate metabolic pathways responsible for restoring homeostasis. Existence of such functional gene clusters points to the possible role of epigenetic regulation in medium- to long-term adaptation to specific types of stress.

## 2. Epigenetic Adaptation and Transgenerational Epigenetic Inheritance

Early observations in plants suggest that stress can cause whole genome changes such as large-scale recombination, chromosomal breakage-fusion cycles, and loss of chromosomes that would lead to genome remodeling with likely phenotypic changes [14]. These global genomic changes are mostly induced and facilitated by reactivated mobile genome elements [15] through relaxation of epigenetic mechanisms, such as repressive covalent modifications to DNA and histone proteins which normally keep them in the silent heterochromatic state [16, 17]. There is an excellent example of association between the chromosome breakpoints and hypomethylation of repetitive (Alu) elements in genomes of white-cheeked gibbons [18], possibly explaining the increased rate of chromosomal rearrangements in the gibbon species. In all other cases, mammalian genomes were found to be remarkably stable. In humans, incorrect recombination events and chromosome rearrangements may lead to cancer, which is in most cases characterized by global genome hypomethylation.

Organisms are able to regulate genome activity in response to stress through specific gene-environment interactions mediated by epigenetic mechanisms, which include DNA methylation, histone modifications, chromatin remodeling activities, and action of various small noncoding RNAs. Abundant evidence suggests that epigenetic pathways are not only targets of stochastic malfunction caused by stress but also parts of the programmed adaptive responses ([19, 20]; see for reviews). The epigenetic stress response can be short term, in which case the relaxation of epigenetic mechanisms (mostly histone modifications) is transient and followed by restoration of the original state as the stress factors are removed [21].

We are, however, interested in the gene-environment interactions mediated by epigenetic mechanisms that result in long-lasting effects. Epigenetic marks are particularly sensitive during the early stages of development, at which point they are influenced by factors such as nutrition, toxins, mother's behavior, and stress. The events form this early life can be strong predictors of adult phenotype, that is, pathological phenotype [19, 20]. Although some long-term consequences of early environmental influences can include impaired development resulting from incomplete buffering against stress [22], there are many epigenetic examples for early-life developmental plasticity which results in long-lasting adaptive phenotypes [20, 23, 24]. In addition, evidence is accumulating for meiotic stability of some of these marks, which obviously can escape two waves of epigenetic reprogramming events—one during the formation of gametes and another shortly after fertilization [25]. The potential of altered epigenetic signatures to be transmitted to the next generations through germ-line cells provides a role for epigenetics in (micro)evolution. Recent study of Zybel and coworkers has shown multigenerational adaptation of hepatic wound healing through specific epigenetic alterations that occurred in sperm, that is, changes in cytosine methylation, the polycomb mark H3K27me3, and the variant histone H3A.Z in promoters of the two genes, PPAR-*γ* (the master transcriptional repressor of hepatic stellate cells) and TGF-*β*1 (the major autocrine and paracrine fibrogenic growth factor) [26]. Transgenerational epigenetic inheritance is an exciting topic in the epigenetic field, with evidence for this phenomenon accumulating for plant and animal organisms as well as for humans [18, 26–35].

The role of transgenerational epigenetic inheritance in evolution has been discussed by Jablonka and Raz in their extensive review [36], where they explicitly mention epigenetic change guiding the selection of genetic variants. They build upon an old idea that such selection can lead to a change from stimulus-dependent to constitutive phenotypic response, which is adaptive because it represents fixation of the response to prevalent environmental conditions—the so-called “genetic assimilation” (for a more recent discussion of that topic, see [37]). Such crosstalk between the epigenome, which reflects intracellular metabolic adaptation to the environment, and the genome, with its stable inheritance, represents an essentially neo-Lamarckism concept, for which a molecular mechanism can be envisioned.

## 3. Neo-Lamarckist Evolutionary Implications

The Lamarckian concept of evolution assumes the direct influence of the environment on the heritable traits of an organism. This view of evolution and the underlying driving forces has been superseded by the well-known principle of evolution by natural selection. However, considering the role of epigenetics in adaptation—especially the long-term epigenetic adaptation—we can envision a scenario where the environmental factors directly shape heritable traits of an organism.

The notable example of aphids producing a larger proportion of winged offspring when exposed to the alarm pheromone [38] clearly demonstrates a direct epigenetically mediated influence of the environment on the next generation—the winged aphids are better equipped to evade a source of stress sensed by their parents. The recent outstanding report of viRNA- (small interfering, virus-derived) mediated viral silencing in *C. elegans* that persists over 5 generations, induced by exposure of the animals to a specific, biologically relevant physiological challenge [34], captures the Lamarckian concept of inheritance of an acquired trait, that is, antiviral response in this case. However, though able to persist for several generations, such epigenetically maintained traits are ultimately unstable, and if a change in a heritable trait is not permanent, it cannot be considered an evolutionary driving force. A full cycle of Lamarckian-type evolution would require either the integration of the acquired environmental information into the genome or its permanent transgenerational epigenetic inheritance, for which there is currently no evidence.

**Figure 1:**
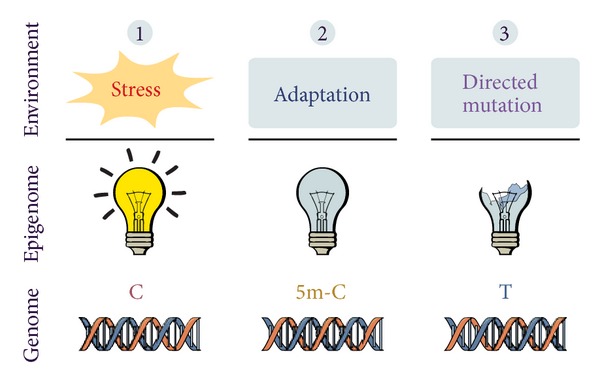
Lamarckian aspects of epigenetic adaptation. An active regulatory gene responds to environmental stress (1) by changing its expression profile to inactive (2), which is mediated by an epigenetic change—methylation of cytosine to 5-methylcytosine. This change increases the mutation potential of the affected genome region. The longer the gene stays inactive and methylated, the higher the chance for a mutation of 5-methylcytosine to thymine, possibly accompanied by the loss of gene function (3). According to this model, long-term adaptation to an environmental influence can be permanently recorded in the genome. The described directed shaping of stably inherited traits by an environmental influence and the accompanying adaptation is a distinctly Lamarckian concept.

Yet, we can envision a way to permanently record the ephemeral epigenetic adaptive change in the genome, although the explanation stays speculative (Figure 1). The most probable mechanism for long-term epigenetic change and transgenerational inheritance is methylation of genes and gene promoters and/or epigenetic control regions [35]. While cytosine methylation is involved in regulation of gene expression, methylation also represents a chemical modification to the cytosine nucleotide (5-methylcytosine, 5-mC), which provides it with a higher potential for mutation. Specifically, densely methylated regions, such as CpG islands within gene promoters, are potentially highly mutable—a transition of 5-mC to thymine, which is inefficiently repaired by the methyl-binding domain 4 (MBD4) protein with a thymine DNA glycosidase activity, would lead to a C-T transition. This transition represents one-third of all point mutations in the human genome and is the most common reason for single cause human diseases [39]. A similar molecular mechanism potentially connecting epigenetic and genetic information has been discussed as another face of the action of activation-induced cytidine deaminase, which has an established role in the maturation of B-cells and somatic mutations in their Ig gene variable regions [40].

It is tempting to speculate that an increase in the mutation rate of an epigenetically silenced (hypermethylated) gene might open a window for a permanent record of an epigenetic change in the genome. Assuming that the epigenetic change was an adaptive response to the changing environment, an increase in the mutation rate of the affected gene could be seen as the missing link in the chain of events needed to connect an external influence with a permanent adaptive change in the genome. The probability for such an event would depend on the time the gene (or its promoter) spends in the methylated state, thus linking the mutation rate with the duration of the external stimuli leading to epigenetic adaptation. The idea that epigenetically regulated regions have a higher susceptibility to mutation, which can drive evolution, has been discussed previously [36], although primarily in the context of chromatin remodeling and without proposing a molecular mechanism directly linked to methylation.

The existence of such proposed “Lamarckian cycle” has yet to be supported by experimental or observational evidence. While confirming its possible role in evolution would be very interesting from the conceptual and the theoretical standpoint, its quantitative contribution as an evolutionary force would in any case be limited in both magnitude and scope, completely overshadowed by—and secondary to—the classical natural selection, to which it would relate as a directed mutation-generating mechanism.

Mutations arising by the proposed mechanism would be unique in being guided directly by epigenetic adaptation to produce a more fit phenotype, representing a form of genetic assimilation [37]. The genes switched “on” by methylation during adaptation to prevailing environmental conditions would be permanently activated by mutation, resulting in the adapted state being stably transmitted in the genome. The loss-of-function type of mutation fixating the “off” state of a gene (i.e., achieved by cytosine methylation) could act on a repressor, which might be a master regulator of an appropriate adaptation pathway, therefore logically inverting the inactive state of the switch to activation of a gene cluster encoding components of a stress response pathway. Direct mutational fixation of an “on” state (i.e., achieved by cytosine demethylation) of a gene cannot be envisioned within the proposed model.

## 4. The Adaptive Response Continuum

**Figure 2:**
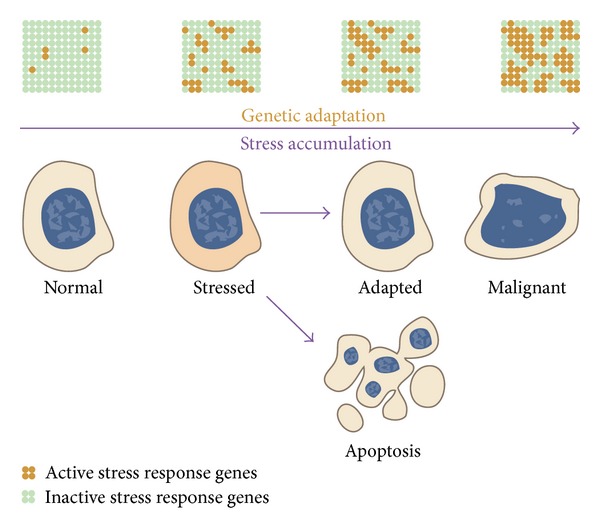
The continuum of adaptive deregulation in response to stress. A cell experiencing stress conditions responds by activating the appropriate gene clusters. As stress accumulates, cells which avoid apoptosis respond with further epigenetically mediated adaptation, which includes deregulation of expression in an attempt to activate progressively more response gene clusters, thus restoring homeostasis. Finally, progressive deregulation weakens cellular control mechanisms, which facilitates malignant transformation. The expression profile of a highly adapted cell shares many similarities with a malignantly transformed cell, which can be seen as a result of extreme adaptation.

In the previous sections, adaptive cellular response to stress has been explained in terms of activation of genes for several metabolic response pathways, each of them responding to several seemingly unrelated but fundamentally connected sources of stress. Arguments for the pivotal role of epigenetics in long-term adaptation and transgenerational inheritance have been presented. At this stage, we can introduce the adaptive deregulation of the epigenome in response to stress (ADERS) hypothesis (Figure 2).

It is safe to assume that minor disruptions of cellular processes, brought about by low levels of stress, are quickly dealt with by the appropriate mechanisms, which can restore homeostasis without significant damage to the cell or long-term consequences for cellular function or integrity. However, as the stress level increases or stress becomes constant, more adjustments are necessary to achieve the same goal, which means that activity of more genes (or whole metabolic pathways) needs to be adjusted. We can define accumulated stress as any intense, repeated, or long-term stress—the key being prolonged duration and relatively high intensity—enough to cause a measurable disruption of cellular processes over a time which is long enough to necessitate long-term adaptation.

Stress accumulation is mirrored in the accumulation of adaptive responses—it is not the departure from homeostasis (which must be dealt with quickly) but the long-term activation of stress response pathways that defines the “accumulated” stress.

In response to stress accumulation, a cell traverses the adaptive response continuum (Figure 2). At low stress levels, response mechanisms are only transiently activated. In case of prolonged duration of stress conditions, the changes may be fixed by epigenetic means as long-term adaptation. Continued or intensified stress will elicit an adaptive response in the form of activation of progressively more response mechanisms, activating many stress-response clusters at the same time. Features of this continuum are key to understanding how progressive adaptation can eventually lead to malignant transformation. Those universal features of epigenetic changes under cellular stress can be interpreted as a “panic mode”, in which a cell attempts extreme measures in order to cope with extreme conditions. Beyond a certain limit, the risk of malignant transformation due to deregulation becomes unacceptably high, at which point a disturbed cell normally undergoes apoptosis, triggered by other damage-sensing mechanisms.

The apparent epigenetic deregulation can be quantitatively measured as the increase in Shannon entropy (linked to loss of information content) of the epigenome—a feature shared by stress, aging, and cancer, as discussed by Hannum and coworkers in their recent study [41]. In this way, loss of information by epigenetic deregulation can be seen as another unifying feature of many degenerative processes, thus elegantly linking stress, aging, and cancer.

Above a certain level of accumulated stress, epigenetic changes become the primary factor in coordinating adaptive gene expression [42–44], thus mediating medium- to long-term adaptation. As evidenced by several studies in plants and animals [45], such epigenetic changes tend to be global [46–49] and relatively stable over time [38, 48–54]. They generally follow the pattern of global hypomethylation [44, 55, 56] and specific hypermethylation of specific gene promoters [24, 43, 57] (although there are exceptions), which is a characteristic of responses to many different sources of stress—from pathogens in plants to heavy metal toxicity [46, 57] or oxidative stress in animals [55, 58]. The cell seems to increase its chance for survival by relaxing certain epigenetic control mechanisms [21, 59, 60]. It seems plausible that this mechanism represents an attempt to restore homeostasis by activating metabolic pathways with a more profound influence on cellular physiology. However, the need for a strong response also brings the risk of unleashing a potentially catastrophic vicious cycle of deregulation ultimately leading to genome instability and malignant transformation [60].

Indeed, epigenetic changes resulting from exposure to accumulated stress (global hypomethylation and specific hypermethylation) show a striking similarity to changes found in many types of cancer [61] and seem to be a hallmark of both. Silencing of proapoptotic genes has been documented in a study on arsenic-induced carcinogenicity [60], which is very illustrative because it documents a stress-induced shift in gene expression ultimately leading to cancer—a common scenario also described by other researchers [46, 62]. A proapoptotic gene, which is epigenetically silenced, promotes the survival of both a severely damaged and a malignantly transformed cell [62, 63]. Recent literature gives plenty examples for the role of epigenetic changes in cancer [56, 63–65], as well as their role in adaptive response to stress [24, 43, 44, 59, 66]. It is therefore not surprising to find malignant transformation as an outcome at the far end of the epigenetic stress response continuum.

In summary, the ADERS hypothesis states that malignant transformation (along with some other pathologies) is facilitated by epigenetically mediated extreme adaptation to accumulated stress. Striking similarities between epigenetic profiles of cells surviving severe stress and cancer cells speak in favor of the hypothesis.

## 5. Testing the Hypothesis

Any viable hypothesis needs to be testable in practice; therefore, we propose a plausible way to do this. The main type of experimental data needed is transcription (microarray) and methylation (methylation chip) datasets from different cell types subjected to various types of stress. While such experiments only require common equipment and well-established procedures, many of the needed datasets can be found in publicly available databases of transcription profiling and methylation experiments.

A systematic approach to testing the ADERS hypothesis using expression and methylation data would necessarily involve the following steps: (i) demonstrating a global genome response to various types of stress, (ii) showing that accumulated stress triggers epigenetic changes which promote long-term adaptation by activating the global stress response, (iii) with the response increasing as stress accumulates, manifested by epigenetically mediated activation of progressively more stress response gene clusters, and finally, (iv) a link needs to be established between the epigenetic deregulation and pathology, especially malignant transformation.

A global response to different types of stress, an idea already supported by some experimental evidence and articulated in the gatekeeper hypothesis [1], would alone merit a research in which it would be clearly identified as a universal principle. Genes or gene ontologies differentially expressed between the stress-treated and the control groups could be compared for classes containing defined stress types, possibly yielding a manageable set of patterns, each being characteristic of several classes of related stress sources.

Demonstrating long-term epigenetically mediated adaptation to accumulated stress would require a similar approach—gene expression profiling. Long-term adaptation could be demonstrated by retention of a key part of the expression profile acquired under stress [35].

Carefully following the progression and the development of an established transcriptional response to a defined type of stress from essentially zero to a level defined as accumulated stress could give insight into various stages of gene (in)activation. Analyzing DNA methylation at selected time points would give insight into the time point at which epigenetic deregulation becomes operational, thus clarifying the role of epigenetics in adaptation. Discovery of a temporal pattern of gene (or gene ontology class) expression would be in line with the proposed hypothesis, provided it could be demonstrated that progressively more stress-response clusters are activated as stress is accumulated.

To link expressional deregulation to pathology, one would need to identify the genes involved in disease—particularly malignant transformation—which are differentially methylated at the later stages of stress accumulation. Alternatively, it might suffice to show that the deregulated gene expression and changed methylation patterns have indeed appeared as a result of stress accumulation. There is already sufficient evidence connecting stem-cell-like expression profile with many types of cancer [63].

## 6. Conclusion

We propose the adaptive deregulation of the epigenome in response to stress (ADERS) hypothesis, which considers epigenetically mediated deregulation of transcription under accumulated stress as a means by which cells attempt to restore homeostasis. Finding the right balance between activating many types of adaptive responses and maintaining tight control over gene expression—both factors dependent on the level of transcriptional deregulation—is the key for maximizing adaptability to environmental influences while minimizing the threat of malignant transformation or other pathologies. Proving the hypothesis would give insight into pathological processes—especially malignant transformation—and their relationship to various types of stress, possibly highlighting novel approaches to treatment of disease. Evolutionary aspects of epigenetic inheritance warrant further investigation on their own merit.
